# The Multisensor Array Based on Grown-On-Chip Zinc Oxide Nanorod Network for Selective Discrimination of Alcohol Vapors at Sub-ppm Range

**DOI:** 10.3390/s19194265

**Published:** 2019-10-01

**Authors:** Anton Bobkov, Alexey Varezhnikov, Ilya Plugin, Fedor S. Fedorov, Vanessa Trouillet, Udo Geckle, Martin Sommer, Vladimir Goffman, Vyacheslav Moshnikov, Victor Sysoev

**Affiliations:** 1 Department of Micro- and Nanoelectronics, St. Petersburg Electrotechnical University “LETI”, 197022 St. Petersburg, Russia; darklord125@mail.ru (A.B.); vamoshnikov@mail.ru (V.M.); 2 Physico-Technical Institute, Yuri Gagarin State Technical University of Saratov, 77 Polytechnicheskaya str., 410054 Saratov, Russia; alexspb88@mail.ru (A.V.); ilyaplygin@mail.ru (I.P.); f.fedorov@skoltech.ru (F.S.F.); vggoff@mail.ru (V.G.); 3 Skolkovo Institute of Science and Technology, Skolkovo Innovation Center, 3 Nobel str., 121205 Moscow, Russia; 4 Institute for Applied Materials, Karlsruhe Institute of Technology, Hermann-von-Helmholtz-Platz 1, 76344 Eggenstein-Leopoldshafen, Germany; vanessa.trouillet@kit.edu (V.T.); udo.geckle@kit.edu (U.G.); 5 Karlsruhe Nano Micro Facility, Karlsruhe Institute of Technology, Hermann-von-Helmholtz-Platz 1, 76344 Eggenstein-Leopoldshafen, Germany; 6 Institute of Microstructure Technology, Karlsruhe Institute of Technology, Hermann-von-Helmholtz-Platz 1, 76344 Eggenstein-Leopoldshafen, Germany; martin.sommer@kit.edu

**Keywords:** zinc oxide, nanorod, gas sensor, multisensor array, selectivity, sensitivity, ethanol, isopropanol, butanol

## Abstract

We discuss the fabrication of gas-analytical multisensor arrays based on ZnO nanorods grown via a hydrothermal route directly on a multielectrode chip. The protocol to deposit the nanorods over the chip includes the primary formation of ZnO nano-clusters over the surface and secondly the oxide hydrothermal growth in a solution that facilitates the appearance of ZnO nanorods in the high aspect ratio which comprise a network. We have tested the proof-of-concept prototype of the ZnO nanorod network-based chip heated up to 400 °C versus three alcohol vapors, ethanol, isopropanol and butanol, at approx. 0.2–5 ppm concentrations when mixed with dry air. The results indicate that the developed chip is highly sensitive to these analytes with a detection limit down to the sub-ppm range. Due to the pristine differences in ZnO nanorod network density the chip yields a vector signal which enables the discrimination of various alcohols at a reasonable degree via processing by linear discriminant analysis even at a sub-ppm concentration range suitable for practical applications.

## 1. Introduction

Environment control in real time requires miniature detector units that deliver a reliable gas-selective signal [1]. So far, the most employed gas sensors able to meet this task are of chemiresistive (or conductometric) type, which—along with electrochemical and thermocatalytic sensors—are the cheapest and easiest to operate [2,3]. These sensors have been available since the 1960s [4] and are now widely used [5] to detect mostly reducing gases under various applications. The basic design of the sensors constitutes a substrate that carries a two-electrode terminal and a chemiresistive material between the electrodes. The most popular materials to develop chemiresistors are wide-gap semiconductors of n-type, mostly oxides, among which the ZnO is possibly the best known one (see Reference [4]) together with SnO_2_ and TiO_2_, to mention a few. The fundamental principle of these ZnO gas sensors is the gas interaction with the oxide surface which results in a change of its resistance with sign depending on reducing or oxidizing nature of the chemisorbed species due to electron exchange between conductance band and at-surface local energy states in the gap. While the efficiency of the chemisorption depends on a density of available chemisorption cites at the oxide surface and their activation (frequently called as a receptor function) the integral resistance change depends valuably on how the at-surface electron exchange affects the total number of free carriers and their transport in the oxide bulk (frequently called as a transducer function). The transducer function is greatly determined by the geometry of the oxide structures: if their effective thickness is less or comparable to Debye length at the surface region the surface chemisorption processes fully manage the overall oxide resistance and we anticipate the most sensitive detector. Because this length falls into a nanometer dimension range [6,7,8] all the investigations on research and development of state-of-the-art sensors based on ZnO consider utilizing nanostructures and nanotechnologies able to produce these structures; for recent reviews see, for instance, References [9,10,11,12].

There are three kinds of ZnO nanomorphology to fit the conditions of matching the Debye length as three-dimensional (3D) nanoparticle layer, (quasi-)two-dimensional (2D) sheets and (quasi-)one-dimensional (1D) nanorods (NRs). Recently, the interest shifted to the latter because of easy production at the large scale and overall stability of properties of such a material [13,14,15,16]. Therefore, plenty of methods and protocols are suggested to grow zinc oxide in the NR morphology like sputtering [17], molecular beam epitaxy [18], atomic layer deposition [19], electrochemical/electrodeposition [20,21,22], colloidal chemistry [23], vapor–liquid–solid deposition [24], sonochemical process [25], etc. However, still, the most convenient and cost-effective method to produce the ZnO NRs at large arrays is hydrothermal one [26,27] based on the crystallization of the material from a solution usually under advanced temperatures and pressure. This technology provides rather good control over the ZnO nanostructure morphology via seeding clusters and managing the growth temperature. Therefore, various investigations dealt with ZnO NRs fabricated in frames of hydrothermal technology to develop prototypes of sensors for detecting many gases ranged from rather simple inorganic ones like carbon dioxide [28], methane [29], nitrogen oxides [30,31,32], humidity [33], hydrogen [34,35,36], to complex organic molecules like ethanol [36,37,38,39,40,41,42,43], acetone [44,45], drugs [46] and glucose [47]. As shown in the cited literature, such ZnO NRs appear to be promising gas-sensing material for chemiresistors to detect the gases mostly at tens or hundreds of ppm concentrations in a mixture with background air. However, the gas selectivity remains rather poor (see, for instance, Reference [40]) as known for all oxide chemiresistors and other gas sensors in general [48]. This challenge is addressed in the most powerful degree by combining gas sensors into multisensor arrays [49] and employing pattern recognition algorithms to process the array vector signals [50].

Here, we consider a cost-effective proof-of-concept prototype of a single-chip multisensor array of chemiresistor type employing ZnO NRs grown by hydrothermal approach directly on the chip equipped with multiple electrodes. We show that the multisensor array based on the elements with even pristine fluctuation of ZnO NR network density could provide a powerful vector signal able to distinguish organic vapors of various alcohols, at ppm and sub-ppm range of concentrations, following the signal processing by linear discriminant analysis (LDA).

## 2. Materials and Methods

As a platform for making multisensor-array chip we employed Si/SiO_2_ substrate, 9 × 10 mm^2^, with cathode sputtered multiple co-planar Pt/Ti electrode strips, about 140 µm width, 4 mm length, approx. 1 μm height, with a between-in gap of about 60 µm [51]. The substrate front side was equipped with two Pt/Ti thermoresistive meanders. Four Pt/Ti heating meanders were realized at the back-side as detailed in Reference [52] to control the chip operating temperature and its spatial distribution up to 400 °C, to be limited by power dissipation at the chip via supported electronics.

The chip was wired with Au wires, 38 µm diameter, into ceramic holder equipped with multi-pin socket, 1.27 mm (SMC, Erni, Richmond, VA, USA) and multiple electrical road connections between chip pads and the output pins being reliably passivated from the environment under heating conditions.

**Figure 1:**
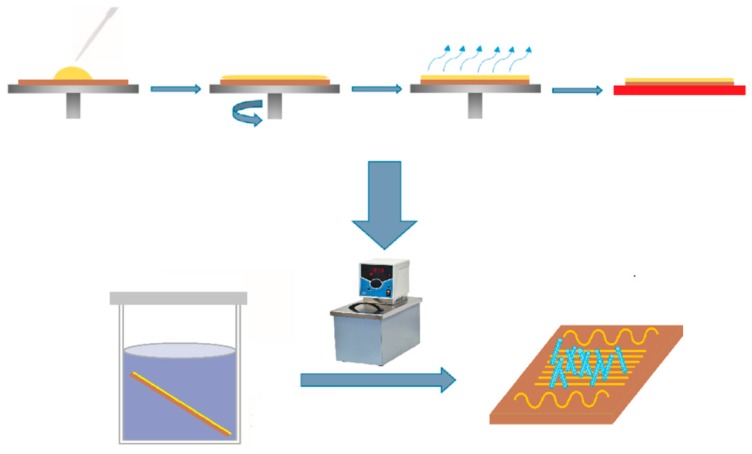
The scheme of ZnO nanorods growth over the multielectrode chip by hydrothermal process. See the text for details.

To grow ZnO NRs layer over the chip we followed a few-step process according to hydrothermal technology (Figure 1) in frames of earlier established protocols [53,54]. All the chemicals used for synthesis were supplied at high purity grade (Vekton). Primarily, we have dissolved Zn(CH_3_CO_2_)_2_ salts in ethanol to get the 5 mM solution which was dropped over the chip substrate and spin coated to get a uniform ZnO seed layer over the surface with grains of ca. 60 nm diameter in average with surface mean density of approx. 21 grains per µm^2^. Then, the chip was dried and annealed at 350 °C for a few minutes in air. We repeated these procedures several times in order to obtain rather thick density of ZnO seed clusters. Further, the chip was placed into a water solution containing a mixture of 10 mM of Zn(CH_3_CO_2_)_2_, 10 mM of (CH_2_)_6_N_4_ and 1 mM of surfactant [(C_16_H_33_)N(CH_3_)_3_]Br to be thermostatically kept at 86 °C for ca. 1 h for facilitating ZnO NR synthesis over seed clusters. Last, the grown ZnO NRs were washed with distilled water and annealed at 350 °C for ca. 30 min to stabilize the structures.

The chip surface area was evaluated by thermal field-emission scanning electron microscopy (FESEM, Carl Zeiss SMT AG, Oberkochen, Germany) combined with energy-dispersive X-ray detector (EDX, Bruker, Quantax 400, Billerica, MA, USA) at 5–15 kV of accelerating voltage. The chemical states at the chip surface were estimated by X-ray photoelectron spectroscopy with a K-Alpha+ spectrometer (Thermo Fisher Scientific, East Grinsted, UK), using a microfocused monochromated Al Kα X-ray source of 50 µm spot size to able measuring at the gap between the chip electrodes. The data were acquired and processed with @Thermo Avantage software, v. 5.9904. In order to avoid a charging effect of the analyzed surface, the K-Alpha+ charge compensation system was utilized via delivering electrons at 8 eV energy and low-energy Ar ions. The obtained XPS spectra were fitted with Voigt profiles under binding energy uncertainty equal to ±0.2 eV. To quantify the elemental composition, the analyzer transmission function, Scofield sensitivity factors [55] and effective attenuation lengths of photoelectrons were taken in attention. All the spectra were referenced versus C 1s peak of hydrocarbon at 285.0 eV binding energy and the energy axis was verified via photoelectron peaks of metallic Cu, Ag, and Au.

To measure the electrical characteristics of the ZnO NR network grown over the multielectrode chip we employed the home-made experimental setup described somewhere [56]. In brief, it was composed of a PC-driven multi-channel acquisition board (NI-6259, National Instruments, Austin, TX, USA) to read out the DC resistances via a low-noise current preamplifier (SR570, Stanford Research Systems, Sunnyvale, CA, USA). The standard DC bias of 5 V was applied to each couple of measuring electrodes, which frame the ZnO NR network segment, in sequence over entire chip by multiplexing card with a rate depending on the resistance value, up to tens of GOhms, to be ordinarily less than 1 sec per segment. Primarily, we tested I–V characteristics of the segments at the range of [−5;+5] V, in forward and back directions, to ensure their linearity and good contact between the oxide NRs and Pt electrodes. The full impedance of the ZnO NR network segments, Z(f), was studied in AC mode with an impedance-meter (NovoControl Alpha AN, Novocontrol Technologies, Montabaur, Germany). Each pair of electrodes of the chip was biased with an AC voltage of 0.1 V and 5.0 V amplitudes as detailed in Reference [57]. The current oscillations at first value of AC voltage could be sensitive to existing energy barriers which are known to be normally up to 1 eV while the second value of AC voltage is equal to the bias utilized in DC mode of resistance measurements. The frequency range was set from 1 Hz to 1 MHz because the frequencies below 1 Hz had significantly enhanced the measurement time to go close to DC mode while we were interested mainly to study a relaxation behavior of the material under high-frequency oscillations to estimate the capacitance contribution. To diminish the possible interference effect of external electric fields, the chip and processing plate circuits were placed into grounded metal box to serve similar to Faraday cage. The operating temperature of the chip was maintained during the electrical measurements to be quasi-homogeneous one by a home-made circuit based on a proportional–integral–derivative controller via adjusting the power dissipated over the back-side heaters to get the accuracy below ±1 °C while the temperature distribution over the chip can be ±10 °C due to intrinsic thermal properties of oxidized Si substrate [52].

**Figure 2:**
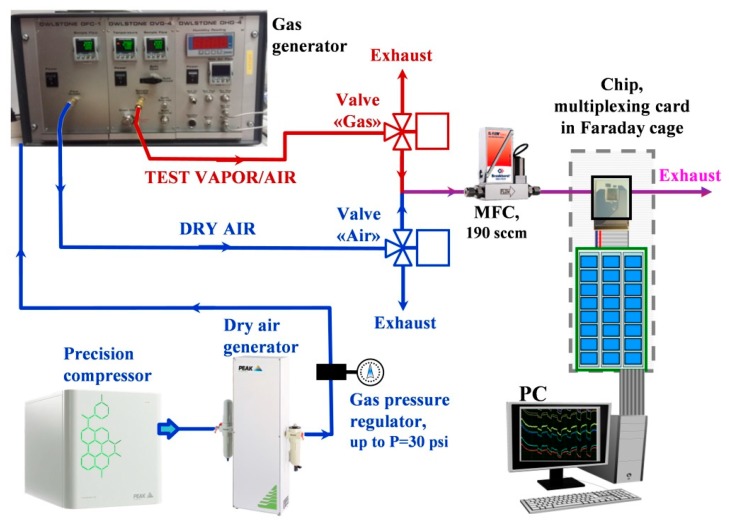
The scheme of experimental setup to measure the gas response of ZnO NR network-based chip. See the text for details.

For gas-sensing measurements, we placed the ZnO NR network-based multielectrode chip into a stainless-steel chamber supplied with input and output tubes with Swagelok^®^ connections to an external gas delivery system. The arrangement of gas entry to the chamber ensured the gas flow to come primarily to the chip surface as detailed elsewhere [58]. As the background gas, we employed lab air which was filtered and dried with the help of dry air generator (PG14L, Peak Scientific, Inchinnan, UK) fed by a precision compressor (Peak Scientific, UK). The whole setup is drawn in Figure 2. The standard flow rate going through the chip at this study was 190 sccm. The background air of almost zero humidity has been enriched with vapors of various alcohols (ethanol, isopropanol, and butanol) as test ones at this research to be evaporated from permeation tubes filled with corresponding liquids [59] using a gas generator (OVG-4, Owlstone, Cambridge, UK). The vapor concentration at the generator output was controlled by its operating temperature and flow rate through the heating chamber containing the permeation tubes. Two valves ensured switching of gas, background air or test vapor mixture, to be delivered to the chip via a high-precision gas mass-flow controller (Bronkhorst, The Netherlands) at the constant rate. We tested the vapor concentrations down to the lowest possible values in the generator at temperatures around 40 °C in sub-ppm range (0.7 ppm for ethanol, 0.4 ppm for isopropanol, 0.2 ppm for butanol) and up to 5 ppm as the highest value for each vapor. The vapors were supplied to the chip for 2 h for each gas concentration with intermediate purging by background air for 3 h as a standard procedure in this study to ensure obtaining equilibrium and stable chip responses in amounts appropriate for signal processing techniques. The vapor response of ZnO NR network segment in the multielectrode chip was calculated according to

(1) $$S=\frac{G_{gas}-G_{air}}{G_{air}}$$

where *G_gas_* and *G_air_* are the segment conductance upon chip exposures to test vapor/air mixtures and to background air, respectively.

To test the vapor selectivity performance of the multisensor array formed on the ZnO NR network-based chip, we collected the resistances of segments recorded upon exposure to background air and vapor/air mixtures as feed vector values to LDA algorithm. This technique is known to extract the classes-related features by funding a maximum of the ratio of between-class to in-class variation when transferring the multidimensional vector signals into a reduced coordinate system [60]. The dimensionality of this coordinate system is equal to the number of classes, in our case, vapors of interest together with the background air as a reference, minus one as discussed in our previous works [61,62]. Different to other powerful pattern recognition techniques, like artificial neural networks [63], this algorithm illustrates clearly the difference between vector signals related to various measuring classes (vapors, in our case); the Mahalanobis distance between their gravity centers in the LDA artificial space could serve as a selectivity measure to distinguish these classes. Here, we employ mainly 2-dimensional LDA diagrams based on the first two LDA components to show the gas differentiation.

## 3. Results and Discussion

### 3.1. The Analytical Characterization of ZnO NR Network

**Figure 3:**
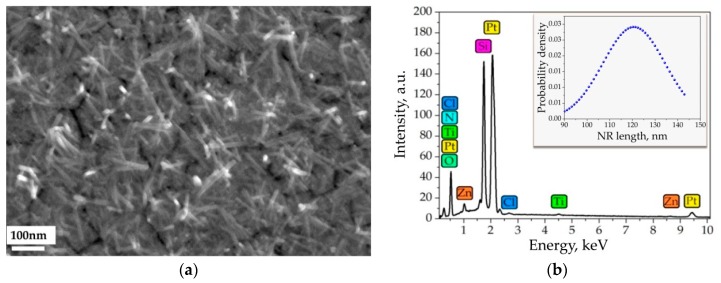
The electron microscopy characterization of the ZnO NRs grown over the multielectrode chip: (**a**) SEM image of the exemplary area of the chip surface; (**b**) EDX spectrum recorded from the chip surface comprising electrodes; the insert shows the Gauss distribution of NR length in the network analyzed from the SEM image.

The SEM studies reveal that the ZnO NRs grow rather randomly in order not to be perpendicular to the substrate and constitute a mesoporous layer (Figure 3a). This structure is a favorite one for gas sensor performance since the surface of the most oxide nanostructures is exposed to the environment. Besides, the NRs contacts multiply each other to provide the proper electronic transport through the NR network. The NR density over the chip surface is intrinsically inhomogeneous from segment to segment in the multisensor array that results in variations of their functional characteristics as discussed in Section 3.3. The diameter of the NRs varies typically from 10 nm to 20 nm while the length goes higher than 90 nm and up to 150 nm (insert, Figure 3b) that results in rather high aspect ratio of these structures. Such geometry in nanodomain also meets the requirement to be comparable with Debye length in non-stoichiometric zinc oxide which should be less than 10 nm under heating the material up to advanced operating temperatures around 300 °C [64]. The EDX spectrum taken from the inspected surface of the chip supports the presence of Zn while other chemical elements are observed, too (Figure 3b). These elements come from electrodes (Pt and Ti), substrate (Si) and some residuals (Cl, N) left after the NR growth.

**Figure 4:**
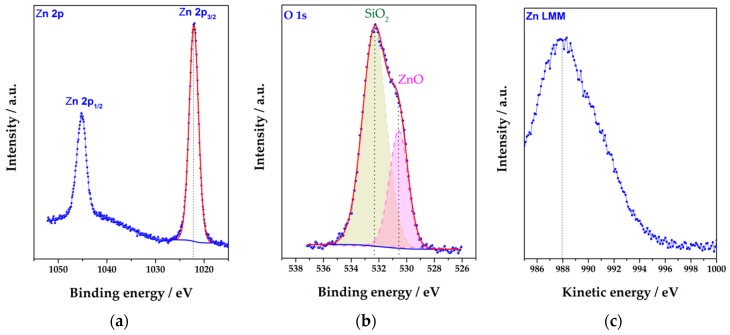
The XPS characterization of the ZnO NRs grown over the multielectrode chip. The chosen energy ranges correspond to Zn 2p (**a**), O 1s (**b**) XP peaks, and Zn LMM Auger line (**c**). Blue points are experimental data; red curves indicate fitting calculations with @Thermo Avantage software.

The data of the XPS characterization of the material at the chip surface are given in Figure 4 in the energy ranges specific for zinc (Figure 4a,c) and oxygen (Figure 4b). The Zn 2p doublet with Zn 2p_3/2_ at 1022.0 eV and Zn 2p_1/2_ at 1045.1 eV cannot alone provide information about the oxidation state of Zn. For a clear identification it is necessary to consider the maximum of the Zn LMM Auger line appearing here at ~988 eV which ensures a presence of ZnO (see Reference [65]). The corresponding O 1s peak is observed at 530.5 eV (Figure 4b). All the peak positions are in a good agreement with literature [66]. Furthermore, the [Zn]/[O] ratio calculated using Zn 2p_3/2_ and O 1s peaks is equal to 1.0 ± 0.1 like expected for ZnO. The intensive O 1s peak observed at 532.3 eV stems from the underlying SiO_2_ substrate surface that supports a mesoporous structure of the ZnO NR network. In addition, this peak overlaps with the possible present surface Zn-OH groups and some surface contaminations also observed in the C 1s spectrum (not shown here).

### 3.2. The Electrical Characterization of ZnO NR Network Under Air Conditions

**Figure 5:**
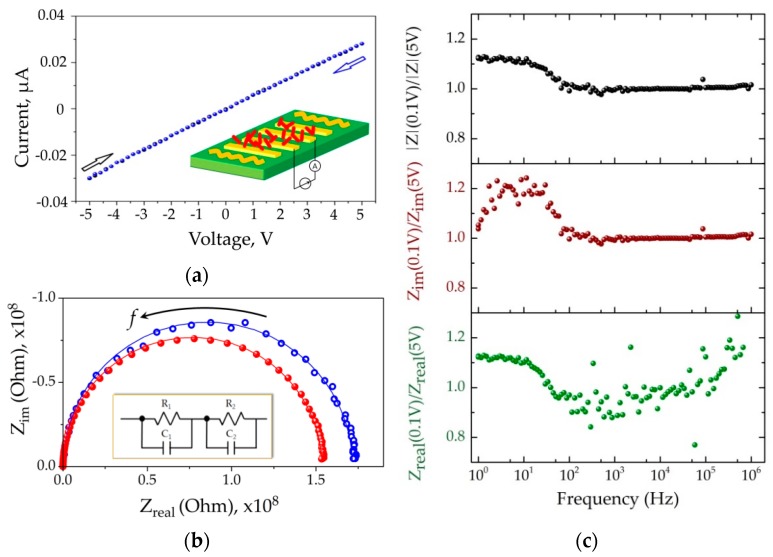
The electrical characterization of the ZnO NRs network at the multielectrode chip in background air conditions under heating to ca. 400 °C. Data for exemplary segment are shown: (**a**) I–V curve taken in DC mode in two opposite direction of electrical potential variation, U_DC_ = [−5,+5] V; insert shows the scheme of measurement; (**b**) Nyquist plot, [1:10^6^] Hz range, the empty blue circles and filled red circles identify the experimental points recorded under U_AC_ = 0.1 V and U_AC_ = 5.0 V, respectively. The corresponding curves going around the points are built accounting for the equivalent electric scheme of the chemiresistors shown in the inset; (**c**) the ratio between full impedance, |Z|, its imaginary, Z_im_, and real, Z_real_, components recorded under U_AC_ = 0.1 V and U_AC_ = 5.0 V in dependence on applied AC frequency.

We have studied the electrical characteristics of the ZnO NR network at the chip surface in DC and AC modes when the chip has been heated up to ca. 400 °C. This temperature corresponds to optimum working one for the chip to observe rather fast and yet maximal chemiresistive performance, as discussed in Section 3.3. Figure 5a shows the exemplary I–V curve taken from a single ZnO NR network segment in the background air. The curve is linear which indicates absence of significant potential barriers in the interface between the ZnO NR network and electrodes.

Figure 5b draws an imaginary part of impedance, Z_im_, versus the real one, Z_real_, of ZnO NR network segments in the AC frequency range from 1 Hz to 10^6^ Hz recorded in the background air. The two spectra are recorded under the magnitude of applied AC electric potential equal to 0.1 V and 5.0 V, accordingly. The obtained data represent a slightly distorted semicircle enabling one to build the equivalent electric scheme of this chemiresistive element which consists of two sequential resistor-capacitor (R-C) circuits. The first R-C component is related to “bulk” conductivity with associated capacitance (at high frequency, see Supplementary Materials, Figure S1), while the second one should be attributed to barriers manifested mainly by junctions existing between the ZnO NRs, being expressed at lower frequencies [67,68]. Indeed, Figure 5c indicates how the bias magnitude affects the impedance, its real and imaginary parts. As one can see, at low electric fields (U = 0.1 V) the impedance at frequencies below 100 Hz is higher than that at U = 5V that appears due to the enhancing of both parts of the impedance. We suggest it reflects the existence of potential barriers at the ZnO NR contacts what impact on both capacitance and resistance of the barriers (the degree of modulation is rather similar).

### 3.3. The Gas-Sensing Characterization of ZnO NR Network Upon Exposure to Alcohol Vapors

**Figure 6:**
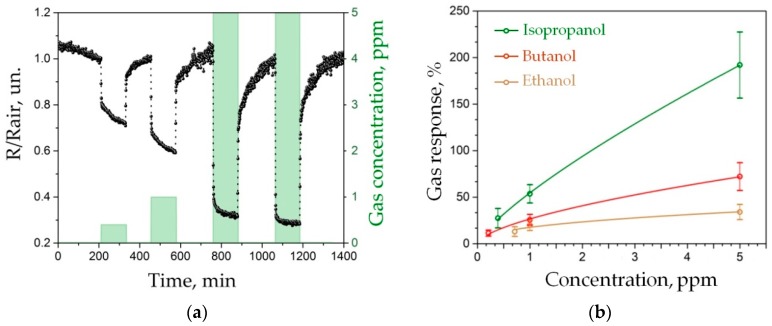
The gas-sensing characterization of the ZnO NR network-based multielectrode chip heated up to ca. 400 °C at DC mode: (**a**) the typical resistance variation of exemplary segment upon chip exposure to isopropanol vapors mixed with air at concentration of 0.4 ppm, 1 ppm and 5 ppm; (**b**) the dependence of chemiresistive response of the segments in the multisensor array to three alcohol vapors on their concentration; the error bar shows the scatter of data over the multisensor array.

We have tested the ZnO NR network-based chip when exposed to alcohol vapors in a range of operating temperatures up to 400 °C, the highest possible temperature, and found in preliminary measurements that this maximum temperature is optimal to observe rather fast and remarkable chemiresistive effect in these nanostructures in accordance with other studies (see, for instance, Reference [69]). Figure 6a gives an example of how the resistance of one of ZnO NR network segments changes when isopropanol vapors appear. The resistance drops in the presence of this reducing gas according to the electronic type of conductivity of this non-stoichiometric oxide. We show the chip exposure to three isopropanol concentrations, from 0.4 ppm to 5 ppm, in mixture with background air. As one can see, the effect is reversible and reproducible because the oxide nanostructures are well stabilized. Similar data have been recorded versus ethanol and butanol vapors. The observed minute response/recovery time of the obtained chemiresistive response still accounts for a gas delivery via the gas-mixing setup. Such a chemiresistive behavior is explained by primary organic vapor adsorption at the NR surface adsorption cites via acid–base reactions with further redox reactions between the organic vapor molecules and chemisorbed oxygen [70]. These processes force the electrons localized by oxygen species at surface region to go back to the ZnO conductance band that increases the bulk conductivity of this n-type material and lowers the potential barriers between NRs [2,3].

We have collected the chemiresistive data for all the ZnO NR network segments of the multisensor array in Figure 6b as the response-to-concentration curves characterizing the three test vapors of ethanol, isopropanol and butanol. The obtained data follow Freundlich adsorption isotherm, $S\sim C^{a}$, where the power index *a* characterizing different alcohols is equal to 0.42, 0.78, 0.62 for ethanol, isopropanol and butanol vapors, respectively, which are rather typical values for chemiresistive oxides. The error bars given at the curves of Figure 6b display the variation of chemiresistive properties of ZnO NR segments in the multisensor array which seems to come from variations of the local density of the nanorod network. The detection limit of the developed sensors is comparable with that of chemiresistive elements based on SnO_2_ nanobelts recorded under similar conditions [71].

**Figure 7:**
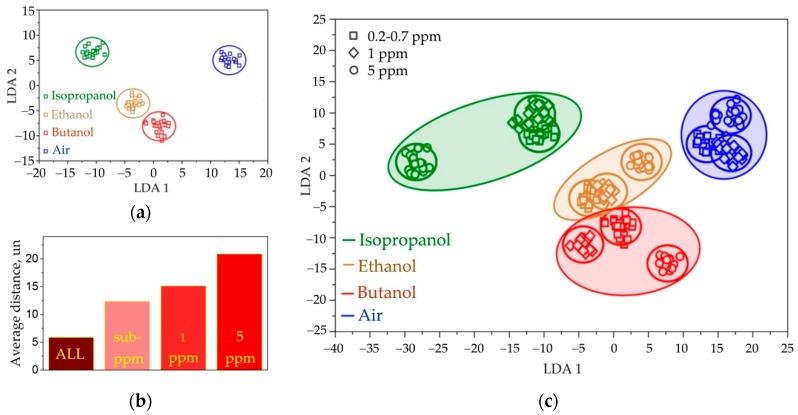
The processing of vector signal generated by ZnO NR network-based multisensor array by LDA: (**a**) the recognition of the signals to vapors at sub-ppm concentrations (0.7 ppm for ethanol, 0.4 ppm for isopropanol, 0.2 ppm for butanol) in mixture with background air, the circles are built around the cluster gravity centers with 0.95 confidence probability based on sampling of 20 training vector points; (**b**) the average distance between vapor-related clusters in LDA space when processing vector signals to vapors at sub-ppm, 1 ppm, 5 ppm, and all-range concentrations; (**c**) the recognition of vapors at all concentrations in range from sub-ppm to 5 ppm, the spheres are drawn to indicate the areas in the LDA space related to test vapors.

As noted above, the ZnO NR chemiresistive segments in the chip are not selective to the kind of alcohol vapors. Therefore, to distinguish the vapors we considered the vector signals generated by the on-chip multisensor array with LDA. Prior feeding the pattern recognition algorithm, all the resistance values of the multisensor array recorded upon exposure to various gases have been divided by median one over the array as detailed in Reference ([71], Supplementary Materials) firstly to reduce the possible drift effects. Primarily, we were interested in selective identifying the test alcohol vapors at a sub-ppm range where the sensor signals are at their lowest. The obtained data are presented in Figure 7a as a projection of the multisensor array vector signals to the plane of the first two LDA components. In order to frame the LDA space into areas which belong to test organic vapors, the circles have been built around the experimental points with 0.95 confidence probability based on sampling of 20 training vector points. As one can see, the point clusters related to different alcohols are well distinguished with the average distance between gravity centers equal to about 12.5 un. When processing separately the vector signals generated by the ZnO NR multisensor array to vapors of higher concentrations, the distance between vapor-related clusters is enlarged up to approx. 21 un. due to higher sensor responses (Figure 7b).

Considering real-practice conditions, the most interesting ability is to selectively distinguish the vapors appeared independent on concentration. Therefore, we processed all the vector signals from the ZnO NR multisensor array recorded upon exposure to three alcohol vapors at the whole concentration range. Such a concentration variation results in the reduction of the average distance between the vapor-related clusters down to 6 un., which is still enough to reliably identify the kind of vapors, as one can see in Figure 7c. In order to distinguish and eliminate the effect of interferences like humidity under practical applications, the multisensor array could be further calibrated as shown previously in Reference [72].

## 4. Conclusions

This study shows that the hydrothermal route is a cost-effective and powerful method to develop multisensor arrays based on ZnO NRs. We found that even pristine variations of NR network density are enough to obtain the gas-specific vector signals which could be employed to distinguish chemically akin organic vapors, like alcohols of various kinds, while the NR morphology allows us to obtain a high sensitivity with a detection limit down to sub-ppm of concentrations. As shown in many other works dealing with ZnO NRs, there is plenty of room for further doping of this material with surface and bulk catalytic elements which could advance both its sensitivity and selectivity to further adjust the characteristics of the multisensor array for specific applications versus gases of interest. The practical aspects of the ZnO NR-based multisensor array performance over a long time will be addressed in further work.

## Supplementary Materials

The following are available online at https://www.mdpi.com/1424-8220/19/19/4265/s1, Figure S1: The high-frequency section of Nyquist plot of Figure 5b.
