# Supplementary material for: The Multisensor Array Based on Grown-On-Chip Zinc Oxide Nanorod Network for Selective Discrimination of Alcohol Vapors at Sub-ppm Range
Source: Sensors (Basel). 2019 Oct 1;19(19):4265. doi: 10.3390/s19194265 (PMC6806624; doi:10.3390/s19194265)
Supplement: Supplementary file 1 [file sensors-19-04265-s001.pdf]

sensors

SUPPLEMENTAL

The Multisensor Array Based on Grown-on-Chip Zinc Oxide Nanorod Network for Selective Discrimination of Alcohol Vapors at Sub-ppm Range

Anton Bobkov[1], Alexey Varezhnikov[2], Ilya Plugin[2], Fedor S. Fedorov[2,3], Vanessa Trouillet[4,5], Udo Geckle[4], Martin Sommer[6], Vladimir Goffman[2], Vyacheslav Moshnikov[1], and Victor Sysoev[2,*]

1 St. Petersburg Electrotechnical University "LETI," St. Petersburg, Russia; darklord125@mail.ru (A. B.), vamoshnikov@mail.ru (V.M.)
2 Yuri Gagarin State Technical University of Saratov, 77 Polytechnicheskaya str., 410054 Saratov, Russia; alexspb88@mail.ru (A.V.); ilyaplygin@mail.ru (I.P.); vggoff@mail.ru (V.G.)
3 Skolkovo Institute of Science and Technology, Skolkovo Innovation Center, 3 Nobel str., 121205 Moscow, Russia; f.fedorov@skoltech.ru
4 Institute for Applied Materials (IAM), Karlsruhe Institute of Technology, Hermann-von-Helmholtz-Platz 1, 76344 Eggenstein-Leopoldshafen, Germany; vanessa.trouillet@kit.edu (V.T.); udo.geckle@kit.edu (U.G.);
5 Karlsruhe Nano Micro Facility (KNMF), Karlsruhe Institute of Technology, Hermann-von-Helmholtz-Platz 1, 76344 Eggenstein-Leopoldshafen, Germany; vanessa.trouillet@kit.edu (V.T.);
6 Institute of Microstructure Technology (IMT), Karlsruhe Institute of Technology, Hermann-von-Helmholtz-Platz 1, 76344 Eggenstein-Leopoldshafen, martin.sommer@kit.edu (M.S.)

* Correspondence: vsysoev@sstu.ru; Tel.: +7-8452-998624

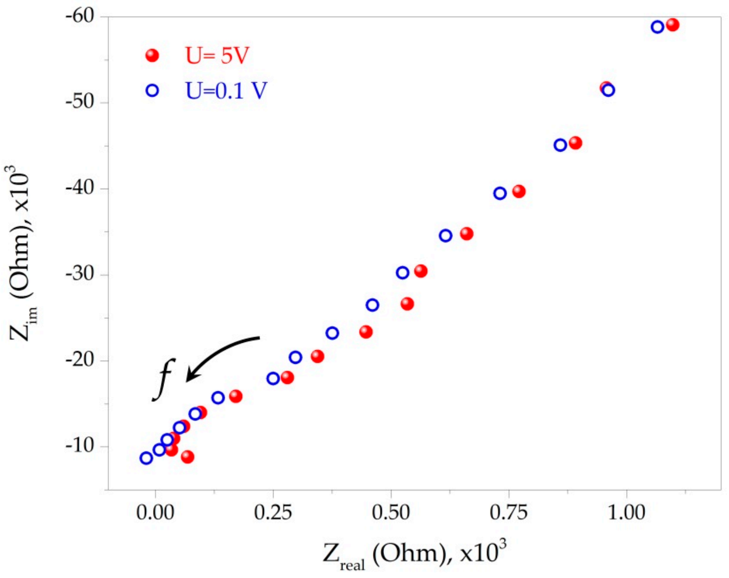

Figure S1. The electrical characterization of the ZnO NRs network at the multielectrode chip in background air conditions under heating to ca. 400 °C. Data for exemplary segment are shown. The high-frequency section of Nyquist plot, Figure 5b of the main text. The empty blue circles and filled red circles identify the experimental points recorded under U_{AC} = 0.1 V and U_{AC} = 5.0 V, respectively.

© 2019 by the authors. Submitted for possible open access publication under the terms and conditions of the Creative Commons Attribution (CC BY) license (http://creativecommons.org/licenses/by/4.0/).
